# Medical Zoology and Mineralogy; or, Illustrations and Descriptions of the Animals and Minerals Employed in Medicine, and of the Preparations Derived from Them, &c. &c. &c.

**Published:** 1831

**Authors:** 


### XIII.

### MEDICAL ZOOLOGY AND MINERALOGY; OR, ILLUSTRATIONS AND DESCRIPTIONS OF THE ANIMALS AND MINERALS EMPLOYED IN MEDICINE, AND OF THE PREPARATIONS DERIVED FROM THEM, &c. &c. &c. By *John Stephenson*, M.D. F.L.S.

THE favourable reception which the work on Medical Botany, lately completed, has experienced, would appear to have given rise to the present undertaking. There can be no question that an acquaintance with Medical Zoology would be highly useful, though not absolutely indispensable, to the medical practitioner, and in the present day of intellectual emulation, we doubt not that opportunities for its acquirement would be eagerly embraced by many individuals. The work whose title is given above appears well calculated to fulfil the indications, and will prove an acquisition to the medical public. Its form, size, and price, are precisely those of the "Medical Botany" to which we have already alluded, and we venture to say that the purchasers of one will be the purchasers of both. It is published in monthly numbers, at a moderate expense, and contains a very large amount of useful knowledge, as well as entertaining information. It cannot be supposed that a formal review of a work of this description is admissible, but we may give one or two extracts in order to shew the style and handling of the matter. Of the five numbers which have now (we are writing in May) seen the light, the last is the best. It contains a description of several varieties of snake and serpent. Take the following description of the Egyptian Asp as a specimen.

"NAJA HAJE.—*The Hajè, Asp, or Aspic.*

SPEC. CHAR. *Body* olive brown, variegated with white; *abdomen* whitish, with blackish spots; *neck* capable of inflation; *length* five to six feet.

Le vipere Haje; *Daudin, Rep.* vi. p. 41. L'Aspic; *Geoffroy, Rept. Egypt.* suppl. t. 3.

THIS species, which has attained more than ordinary celebrity from being supposed to be the animal whose poison the famous Cleopatra selected to terminate her existence, is found abundantly in Lower Egypt, sometimes in hedges, and sometimes in the fields. 'It is universally known,' says Mr. Griffith, 'that this illustrious princess, abandoned by fortune, who had so long smiled upon her, commanded that a reptile of this species should be brought to her, concealed in fruits and flowers, and caused it to bite her, to put a period to her misfortunes. But after the fall of the Roman empire, though Egypt still preserved some traces of the high renown of Cleopatra, and though the name of the Aspic was not pronounced without some degree of horror by all the people of Europe, still for a long series of ages the true species of the serpent was unknown, and the Cerastes, the Egyptian Viper, the Ammodytes,* and the Lebetina, were taken for it. Bruce declared for the first of these opinions, Forskal for the last, and

---

* "The Coluber Ammodytes, *Jacq. Coll.* iv. t. 24, 25, a species greatly allied to the Viper, from which it is distinguished by an erect process or wart at the tip of the muzzle. It is an extremely poisonous reptile, and inhabits the mountainous parts of Illyria."

Laurenti, Hasselquist, Daudin and Count La Cépède, for the second, which undoubtedly has some plausibility, for it is well proved that under the name of ασπις, the ancients were acquainted with many venomous serpents aboriginal of Egypt.

'It has been only since the expedition of the French to Egypt that the true species of the Aspic has been ascertained. During the period of that expedition, the French philosophers attached to the army observed a species of ophidian, regarded as harmless by Linneus and most herpetologists, but considered as extremely venomous by the traveller Forskal. This ophidian is called *hajé* by the inhabitants, and recent travellers have incontestibly proved that it is the true aspic of the ancients, which never inhabited Europe; for the reptile which some years since infested the forest of Fontainbleau, and was called by this name, was nothing but a variety of the common viper; and the *Œsping* of the Swedes, is quite another species from the one in question.'*

The ancients entertained a notion that the poison of this serpent is more deadly than that of any other venomous creature inhabiting the East; that its bite, though inevitably mortal, produced no pain or violent symptoms, and merely occasioned the gradual diminution of pulsation, which was followed within twenty-four hours by a profound sleep terminating in death. Galen assures us, that in Alexandria, to shorten the punishment of criminals condemned to death, they were bitten in the breast by an Asp; and Dioscorides asserts that the wounds occasioned by the bite of this reptile are unaccompanied by any local tumefaction, and that they are so small that they appear to have been made with a very fine needle." 89.

Let us turn from the asp, borne amidst roses to the breast of the Egyptian queen, and contemplate one of those frightful and horrid reptiles, which would seem to be inflicted by God upon the earth, as a scourge on the rest of his creation. The most powerful pens have been employed in the endeavour to depict the terror which these creatures inspire in the Islands of the West. The cophias lanceolatus, or yellow viper, which infests Martinique, St. Lucia, and Beconia, is of this appalling character. By a chance or design equally fortunate and inexplicable, these islands alone can lay claim to the unenviable distinction of possessing the viper in question. In St. Lucia and Martinique, the marshes, the tilled grounds, the forests, the borders of rivers, and the mountains, from the level of the sea to the region of the clouds is peopled by the cophias lanceolatus. They are seen every where, their asylum is the sugar-cane plantation, they lie in ambuscade in bush and brake, and they find their way into the cottage, especially into that of the unhappy negro. Our readers will excuse our making another extract.

"These reptiles possess an activity and vivacity of motion truly alarming. A ferocious instinct induces them to dart impetuously upon passengers, either by suddenly letting go the sort of spring which their body forms, rolled in concentric and superpoised circles, and thus shooting like an arrow from the bow of a vigorous archer, or pursuing them by a series of rapid and multiplied leaps, or climbing up trees after them, or even threatening them in a vertical position.

The effects of the bite of these serpents are in general very terrible, but vary considerably, according to a multitude of circumstances. The tumefaction of the part, which soon becomes livid and gangrenous, nausea, convulsions, car-

* *The Animal Kingdom, described and arranged in conformity with its organization, by* Baron Cuvier, *with additional descriptions by* Edward Griffith, *F.L.S.* vol. ix. p. 382.

dialgia, and an invincible somnolency, are the ordinary symptoms of the action of their poison, which either produce death in the course of some hours, or at most some days, or causes for several years vertigos, paralysis, more or less extensive, phagedenic ulcers of a malignant character, and a variety of other distressing infirmities. It is therefore nowise astonishing that the Trigonocephalus is an object of horror, not only to man but also to animals. The horse trembles and prances violently in its presence; rats scud away at its approach, sending forth cries of terror; birds especially, against which it wages inveterate war, manifest their aversion to it by repeated clamours; and the Loxia *indicator*, by pursuing with its cries, often indicates to man the place of its retreat.

The African races, which form a great portion of the population of Martinique, constantly preserve certain organs of this reptile for talismans, either preservative or hurtful. These are called in the Carib language, *piailles*, and they are always found among the materials of those magical conjurations undertaken by the negroes who are addicted to sortilege.

The severity of the accident produced by the bite of the Trigonocephalus varies, as in the case of other venomous serpents, according to the state of health of the bitten subject, the depth and number of the wounds, the time which has elapsed since the animal made use of its fangs, and, consequently, the quantity of poison which had penetrated into the system. But in all possible cases the help of art is indispensable." 98.

A variety of remedies have been tried and vaunted, which proves that none have any decided or specific power.

"In the *post-mortem* examination of such persons as have died some time after the accident, Dr. Renzger, a German physician, whose experience on these subjects has been considerable, always found the brain and spinal cord partially softened, and a considerable effusion of bloody serum in the cavities of the skull, thorax, and abdomen; the lungs and liver were gorged with blood, and as well as the intestinal canal, exhibited gangrenous spots; the cellular tissue round the wound was sloughy, and, on incision, a great quantity of decomposed blood and sanies escaped from it.

In those cases which do not end fatally the wound becomes inflamed and erysipelatous, the general symptoms gradually disappear, and the disease altogether ceases within from three to eight weeks, under general perspiration and bilious diarrhœa. Sometimes, however, great debility and cachectic appearances remain, and death ensues, after two or three years, under paralysis, mental derangement, or dropsical symptoms. In case of ultimate recovery, the healing of the wound always takes place very slowly; the skin and neighbouring cellular tissue slough, and discharge a great quantity of blood; the margins of the wounds are of a livid colour, and bleed on the least touch; after some time, the sloughs having come away, suppuration begins to take place, but is always of an unhealthy kind; cicatrization hardly ever takes place before some months, or even years; the cicatrix is thin, of a livid colour, and liable to be inflamed or absorbed.

The best method of treatment consists in the excision of the wounded part, and its subsequent scarification and cauterization. If the necessary instruments are not at hand, the wound must be sucked and repeatedly washed with water, lemon-juice, or brandy, as in other cases of poisonous wounds; at the same time it is advisable to put a tight bandage round the limb. Stimulants must be given internally, and the wound is continually, even after the remission of the general symptoms, to be treated with stimulants and antiseptics, otherwise it will become gangrenous. In India, it appears the Tanjore pill, of which arsenic is the chief ingredient, has been exhibited with considerable success against the bites of venomous serpents. In the second volume of the Medico-Chirurgical Transactions, there is a series of cases related by Mr. Ireland, surgeon of the 60th regiment, where arsenic was administered in very *large* doses with good effects. Mr. Ireland, on his arrival in the island of St. Lucia, was informed that an officer and several men belonging to the 68th regiment, had died from the bites of the Fer-de-lance, and that every thing had been tried by the medical men, to no purpose, he was determined to give arsenic a full trial. In these cases he exhibited two drachms of Fowler's solution with ten drops of laudanum, in the effervescing draught, and repeated it every three or four hours. Externally, the parts were frequently fomented with warm water, and rubbed with a liniment composed of oil of turpentine, liquid ammonia, and olive oil." 100.

From the foregoing specimens the professional public may form some opinion on the nature and probable value of the "Medical Zoology." The author, we imagine, makes no pretence to its being much more than a compendium, and this is all that is required. We shall watch the progress of the work, and report upon it from time to time. We hope that Dr. Stephenson will not relax in his endeavours to render it as complete and as accurate as possible. There are many errors of the press in the present numbers, and we would recommend the industrious author not to slumber on his post, but to strain every nerve in the progress of his undertaking. We recommend our readers to procure and patronize the "Medical Zoology."

